# An experimental study of the process of felt understanding in intergroup relations: Japanese and Chinese relations in Japan

**DOI:** 10.1038/s41598-024-63227-0

**Published:** 2024-06-07

**Authors:** Tomohiro Ioku, Eiichiro Watamura

**Affiliations:** Graduate School of Human Sciences, Osaka University, 1-2 Yamadaoka, Suita, 565-0871 Japan

**Keywords:** Felt understanding, Intergroup relations, Felt positive regard, Outgroup stereotypes, Intergroup overlap, Psychology, Human behaviour

## Abstract

“Felt understanding” is a crucial determinant of positive interpersonal and intergroup relationships. However, the question of why felt understanding shapes intergroup relations has been neglected. In a pre-registered test of the process in intergroup relations with a sample from East Asia, we manipulated felt understanding (understood versus misunderstood by an outgroup) in an experimental study (*N* = 476). The results supported the expectation that felt understanding would lead to a more positive intergroup orientation and action intention. The results of parallel mediation analyses showed that felt understanding indirectly predicted intergroup outcomes through felt positive regard, intergroup overlap, and outgroup stereotypes. Furthermore, the results of post-hoc sequential mediation analyses indicated that felt understanding indirectly predicted intergroup outcomes sequentially through felt positive regard and intergroup overlap, followed by outgroup stereotypes.

## Introduction

People feel understood when outgroup members understand their perspectives; this is called felt understanding. In the context of religion, this perception is exemplified in the beliefs of Shias in Lebanon that Sunnis do not understand or accept Shia beliefs. Transgender and gender-diverse people believe that heterosexuals either do or do not understand their orientations. Felt understanding plays an important role in interpersonal and intergroup relationships^1^. However, to the best of our knowledge, no study has clarified why felt understanding is crucial for intergroup relationships. This study addresses this question by experimentally investigating the felt understanding process in intergroup relations. We tested the felt understanding process through an experimental study using a pre-registered design, hypotheses, and analysis.

### Effect of felt understanding in intergroup relations

In intergroup contexts where social identities are prominent, felt understanding is defined as the perception that members of an outgroup understand and accept the perspectives of ingroup members^1–3^. These perspectives include ingroup members’ beliefs, values, experiences, intentions, and identities. For example, the Japanese feel understood when immigrants understand their views in a nonjudgmental manner. According to Livingstone et al.^1^, felt understanding is theoretically related to but distinct from meta-perceptions such as meta-stereotypes. Meta-perceptions are representations of what an outgroup thinks, whereas felt understanding is a representation of what an outgroup thinks about what one thinks. Thus, the latter is a meta-meta-perspective^4,5^. This higher-order perspective is called the third-order intentionality/second-order theory of mind^1,6–8^ making felt understanding unique to intergroup relations.

Livingstone et al.^1^ conducted cross-sectional surveys to provide evidence of the critical role of felt understanding in various intergroup contexts. The results show that the Scottish who believed that the English did not understand their culture supported Scotland’s independence from the UK. Similarly, the British, who thought Europeans did not understand their identity, voted for Brexit in 2016. Furthermore, Catholics in Northern Ireland who perceived that Protestants understood Catholic values tended to trust Protestants. These effects persisted even after controlling for outgroup stereotypes and meta-stereotypes.

In the East Asian context, Ioku and Watamura^9^ cross-culturally replicated the findings of Livingstone et al.^1^. The result of the study found that the Japanese who thought the Chinese understood their perspectives wanted to approach them more positively. After adjusting for multiple outcomes, this effect remained statistically significant. Despite a few cultural differences, possibly due to naïve dialecticism and causal attribution, the overall pattern of results was consistent with that of Livingstone et al.^1^.

These findings were limited to outgroup targets that cohabited with an ingroup in a community with a shared superordinate political structure/system (cohabiting target, e.g., the Chinese in Japan). In contrast, Ioku and Watamura^10^ focused on outgroup targets living in separate communities with different superordinate systems (separate targets, e.g., Chinese in mainland China). As with the Chinese in Japan, when the Japanese believed that the Chinese in mainland China understood the Japanese beliefs, they trusted them. Additionally, the Japanese believed that the Chinese in mainland China understood Japanese beliefs less than those living in Japan. These results suggest that the effect of felt understanding in intergroup contexts can be significant even when the target is a separate entity with a long-standing geopolitical rivalry, whereas the level of felt understanding may differ across cohabiting and separate targets.

Livingstone et al.^11^ conducted multi-experimental studies to provide evidence of the causal effect of felt understanding on intergroup relations. Participants read a fake internet news article stressing that the outgroup either understood or misunderstood the ingroup’s perspectives. In the context of Brexit in the UK, “Leave” voters, who read an article emphasizing that “Remain” voters understood their perspectives, trusted them (as opposed to those who read an article stressing that “Remain” voters misunderstood them). The results of the meta-analysis of the main effects of felt understanding across six studies showed a medium effect size for intergroup orientation (including outgroup trust).

Therefore, there is growing evidence that felt understanding plays a positive role in intergroup relationships. However, previous studies have not investigated the processes through which felt understanding shapes intergroup relations^11^. We believe that at least three central processes are involved: positive regard, intergroup overlap, and stereotyping.

### Process of felt understanding in intergroup relations

First, we argued that felt understanding affected “felt positive regard,” which predicted intergroup outcomes such as outgroup trust. Livingstone et al. ^12^ derived a hypothesis from the literature on positive interpersonal relations^13–15^. Felt positive regard refers to the extent to which an ingroup is positively regarded by an outgroup^12^. This includes feelings of being liked^11,16^ and respected^17–19^. From the perspective of positive regard^13–15^, feeling understood by others satisfies the need for positive regard. Positive appraisal, emotions, and action intentions toward an outgroup are more likely to arise when one feels positively regarded. Livingstone et al.^12^ provided strong evidence for this hypothesis in multiple contexts.

#### Hypothesis 1:

Felt understanding positively affects feelings of positive regard, leading to positive intergroup outcomes.

Second, we expected that felt understanding affected intergroup overlap (or shared identity), which predicted intergroup outcomes. The literature on perspective-taking suggests that perspective-takers attempt to better understand the minds of their targets to understand the world from their standpoint^20,21^. In this sense, taking the viewpoint of others is a psychological merging of minds that results in the perception of “self” and “others” overlap^22^. Goldstein et al.^22^ argued that being on the receiving end and taking another person’s perspective are comparable to two brains that merge and share the same psychological space. If perspective-takers and receivers share such a space, when an ingroup member learns that an outgroup member is taking ingroup perspectives, they will believe that “They are us,” enhancing the sense of intergroup overlap. A growing body of research suggests that intergroup overlap is relevant in intergroup settings^23–25^ and that more overlap can benefit intergroup relations.

#### Hypothesis 2:

Felt understanding positively affects intergroup overlap and lead to positive intergroup outcomes.

Third, we proposed that felt understanding affected outgroup stereotypes, predicting intergroup outcomes. We drew on the theory and research on the norm of reciprocity^26–29^. The norm of reciprocity posits that humans tend to repay favorable treatment and retaliate against unfavorable treatment^26,27^. One helped his peers because they helped him^28^. People engage in incivility when their friends treat them rudely^27,29^. Thus, when ingroup members perceive that outgroup members understand their perspectives, stereotypes may be reduced, particularly negative ones toward an outgroup. Otherwise, negative stereotypes toward an outgroup may be reinforced. Reinforced outgroup stereotypes can harm intergroup relations, and many studies have revealed the detrimental consequences of outgroup stereotypes^30,31^.

#### Hypothesis 3:

Felt understanding negatively affects outgroup stereotypes and leads to positive intergroup outcomes.

While these psychological processes are largely drawn from Western studies, recent findings have broadened the scope to include diverse cultures, such as Lebanon and indigenous areas in Chile^12^. Nevertheless, cultural features should be considered when investigating psychological processes across different cultural and geopolitical settings. For instance, the notion that people must regard themselves positively has been widely embraced^32–34^. However, numerous studies have suggested that the need for positive regard may be weaker, if not largely absent, among East Asian people than among Westerners^35–39^. Thus, it is possible that the weaker need for positive regard in East Asia impedes the process of feeling positive regard. For example, even if the Japanese believe that the Chinese understand their perspectives, they may not feel positively evaluated by the Chinese, because East Asians have a low need to be regarded positively. Moreover, according to naïve dialecticism^9,40,41^, East Asians tend to tolerate contradictions. This tolerance of contradictions may weaken the intergroup overlap process. For example, when the Japanese think that the Chinese understand them, it may not affect their orientations toward the Chinese via intergroup overlap because they believe that Japanese and Chinese people have different perspectives.

### Present study

**Figure 1:**
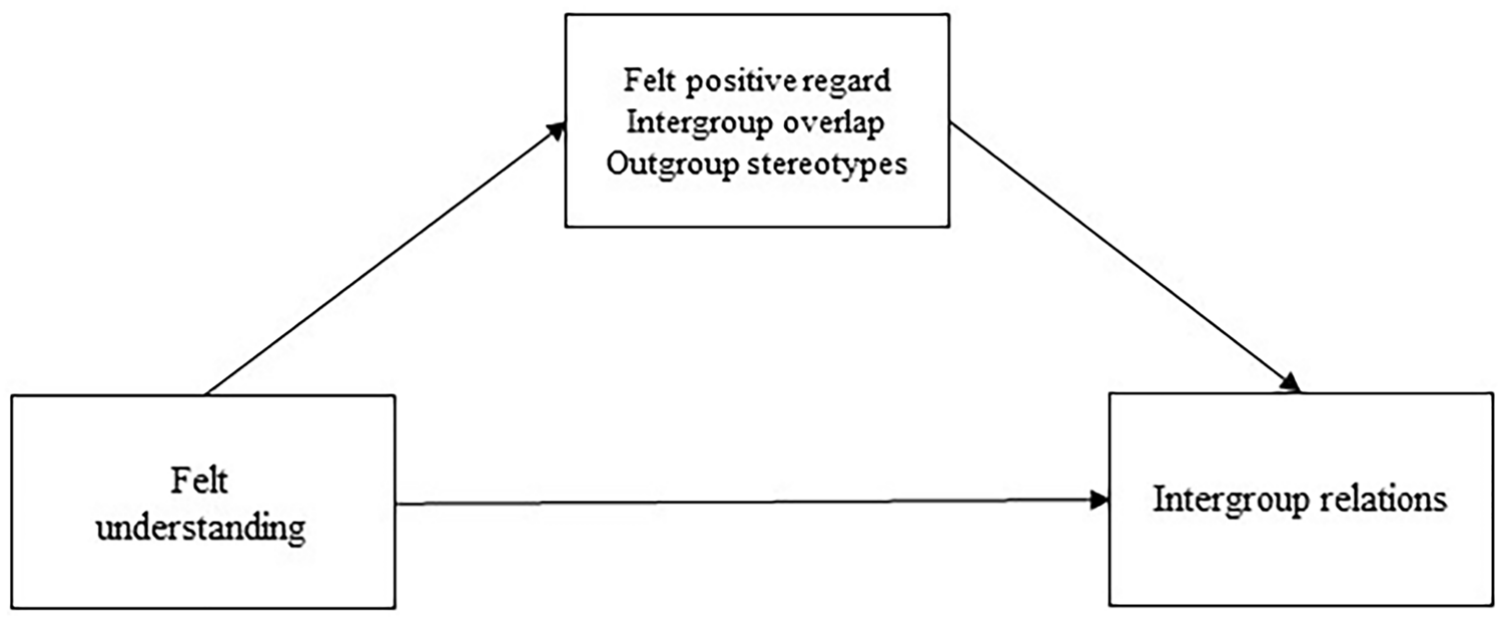
Path diagram for the effects of felt understanding on intergroup relations via felt positive regard, intergroup overlap, and outgroup stereotypes.

As described in the pre-registration protocols, we hypothesized the main effects of felt understanding on intergroup orientation and action intentions. More positive intergroup outcomes were expected in the felt-understanding condition than in the felt-misunderstanding condition. We hypothesized that multiple processes of felt understanding indirectly predicted more positive intergroup outcomes through felt positive regard, intergroup overlap, and outgroup stereotypes. More positive intergroup outcomes were expected to be driven by a greater sense of being positively regarded by the outgroup, a larger perception of overlap between the ingroup and outgroup, and fewer negative stereotypes about the outgroup. Figure 1 illustrates the hypothetical model.

### Study context

This study addresses Japanese and Chinese relations. Compared to Western countries such as the UK, Japan has not yet accepted many immigrants^42^. Despite its relative ethnic homogeneity, Japan faces challenges such as unfavorable public sentiment against migration paired with anti-immigrant political movements^43^. One of the largest immigrant groups, the Chinese, is more likely to be exposed to discrimination and prejudice in the wake of the global pandemic, which is a social issue common in other countries^44–46^. Focusing on Japanese and Chinese relations in Japan has several practical implications.

Furthermore, while Japan shares many economic and political characteristics with Western countries^47,48^, it differs in terms of its need for positive regard and tolerance of contradictions^36,39–41^. As described earlier, the weaker need for positive regard and tolerance of contradictions in East Asia may hinder the process of felt understanding of intergroup relations. Addressing Japanese and Chinese relations in Japan has theoretical implications for felt understanding research in intergroup contexts, since it allows this notion to be explored.

## Method

### Ethical approval

All procedures performed in this study were approved by the Institutional Review Board of Osaka University in Japan and performed in accordance with the relevant guidelines and regulations of Osaka University. Informed consent was obtained from all respondents included in the present study. We pre-registered the hypotheses, design, methods, and analysis plan for this study at https://osf.io/5f8pu/?view_only=bdf6141b7efb45d7bbc7c9c6b5070fe1.

### Participants

We recruited 502 Japanese adults ≥ 18 years old through Yahoo! Cloud sourcing. The recruitment period for this study was May 21–28, 2021. Twenty-six violators of the exclusion criteria were not included (completed the questionnaire but missed 50% or more questions; failed to correctly answer two attention checks, which asked participants to select a “ − 2” on a 7-point scale), leaving a final sample of 476 respondents (302 males, 167 females, and 7 other). The mean age of the sample was 48.17 years (*SD* = 11.24).

### Design

Following the pre-registration plan for this study, we used a between-participants design (felt understanding: understood versus misunderstood). This study measured two sets of dependent variables as in Livingstone et al.^11^. The first was intergroup orientation, which included perceptions of intergroup relationships, feeling thermometers, and outgroup trust. The second factor was action intention, which included approach, avoidance, and confrontation.

### Materials

#### Stimuli

The manipulations in this study were conducted using a fictitious online news article from a media outlet based on Livingstone et al.^11^. Except for minor phrase alterations emphasizing whether the outgroup understood or misunderstood the ingroup’s perspective, the stimulus article was identical across conditions. For example, the news article headline stated that the outgroup’s understanding of the ingroup was either “accurate” (“understood” condition) or “poor” (“misunderstood” condition). The closing quote in the “understood” condition read, “As International Public Opinion representative Kobayashi states: The experience of the Chinese displaying some kind of understanding may explain this Japanese perception.” In contrast, in the “misunderstood” condition, it read, “As International Public Opinion representative Kobayashi states: The experience of the Chinese displaying some kind of misunderstanding may explain this Japanese perception.” Afterward, we asked the participants to describe their thoughts and feelings when they heard about such events.

#### Measures

The following scales were used in this study.

##### Manipulation checks

Felt understanding was assessed using six items. This scale was derived from previous research^1,9^. Example items include “(outgroup members) understand (ingroup members’) values” and “(outgroup members) have a good understanding of what (ingroup members) think.” Responses were given on a scale from − 3 (completely disagree) to 0 (neither) to 3 (completely agree) (α = 0.89).

##### Intergroup orientations: perception of intergroup relationship

Perceptions of the intergroup relationship were assessed using a scale^1,9^ consisting of seven semantically differential items (e.g., negative/positive). These were followed by the phrase, “The relationship between (outgroup) and (ingroup) is….” Participants rated their responses on a 7-point scale from − 3 (negative anchor) to 3 (positive anchor) (α = 0.96).

##### Intergroup orientations: outgroup feelings

Feelings toward the outgroup were measured using a thermometer^11,49^. This ranged from 0° (very cold or unfavorable feelings) to 100° (very warm or extremely favorable feelings) for the outgroup.

##### Intergroup orientations: outgroup trust

Trust in the outgroup was assessed by using six items derived from previous studies^1,9,50^. Example items included statements such as “Most (outgroup members) try to be fair” and “Most (outgroup members) cannot be trusted to act in the interests of (ingroup members).” Responses were recorded on a scale from − 3 (completely disagree) to 0 (neither) to 3 (completely agree) (α = 0.79).

##### Action intentions: confrontation, avoidance, and approach

Action intentions toward the outgroup were measured using ten items adapted from previous research^9,11,30^. These included a 3-item scale of intentions to confront the outgroup (α = 0.84), a 3-item scale of intentions to avoid the outgroup (α = 0.94), and a 4-item scale of intentions to approach the outgroup positively (α = 0.93). Responses were recorded on a scale from 1 (not at all) to 7 (completely).

##### Mediators: felt positive regard

Felt positive regard was measured using a 6-item scale based on prior research^1,11^. These include “outgroup members don’t like ingroup members” (reverse scored) and “outgroup members act favorably toward ingroup members.” Responses ranged from − 3 (completely disagree) to 3 (completely agree) (α = 0.88).

##### Mediators: intergroup overlap

Intergroup overlap was assessed using a pictorial measure, based on previous studies^11,51–53^. This measure represents the ingroup as a circle and the outgroup as another circle positioned along a horizontal line. There were seven versions of this image, with the circle positions ranging from opposite ends of the line (greatest distance, scored as 1) to almost entirely overlapping (least distance, scored as 7). Participants were asked to choose the image that best reflected the relationship between the ingroup and the outgroup.

##### Mediators: outgroup stereotypes

Outgroup stereotypes were assessed using six shorter-item versions of semantic differential scales derived from prior research^1,9^. These items represented the valence of the stereotypes of the outgroup. The list is preceded by the statement “outgroup members tend to be….” Participants rated their responses on a 7-point scale from 1 (positively anchored scale end) to 7 (negatively anchored scale end) (α = 0.93).

### Analyses

Following the pre-registration plans, an overall analysis technique was used to test the hypotheses and estimate the effect sizes. Before testing our hypotheses, we confirmed the intended effect of the felt understanding manipulation using an analysis of variance (ANOVA). Second, we tested the reproducibility of the results of previous studies using a one-way (understood vs. misunderstood) multivariate analysis of variance (MANOVA) on the intergroup orientation variables (perception of the intergroup relationship, outgroup feelings, and outgroup trust) and action intention variables (confrontation, avoidance, and approach). Finally, the hypotheses were investigated using parallel mediation models. In parallel multiple mediation models with 5000 samples and a 95 percent confidence interval (following a recommendation from a reviewer, we changed the pre-registered 2000 samples to 5000), we tested (1) the total indirect effect of felt understanding on intergroup outcomes via felt positive regard, intergroup overlap, and outgroup stereotypes; (2) the specific indirect effect of felt understanding on intergroup outcomes via felt positive regard; (3) the specific indirect effect of felt understanding on intergroup outcomes via intergroup overlap; and (4) the specific indirect effect of a condition on intergroup outcomes via outgroup stereotypes. Although not pre-registered, we ran post hoc sequential mediation models (based on reviewer recommendations) to test the sequential specific indirect effect of felt understanding on intergroup outcomes via felt positive regard and intergroup overlap, and then outgroup stereotypes.

## Results

Table 1 reports the descriptive statistics and zero-order correlations between all variables.

**Table 1:** Zero-order correlations and descriptive statistics for all variables.

	Variable	*M*	*SD*	1	2	3	4	5	6	7	8	9
1	Felt understanding	3.89	1.19									
2	Perception of relationship	3.63	1.24	0.683**								
3	Outgroup trust	3.74	0.97	0.717**	0.735**							
4	Outgroup feeling	43.70	23.42	0.624**	0.731**	0.740**						
5	Avoidance	3.86	1.70	– 0.702**	– 0.687**	– 0.773**	– 0.723**					
6	Approach	3.87	1.39	0.648**	0.645**	0.745**	0.679**	– 0.814**				
7	Confrontation	2.24	1.19	– 0.202**	– 0.202**	– 0.243**	– 0.174**	0.295**	– 0.182**			
8	Intergroup overlap	3.34	1.62	0.535**	0.520**	0.536**	0.553**	– 0.570**	0.523**	– 0.109*		
9	Felt positive regard	3.93	1.03	0.711**	0.702**	0.739**	0.715**	– 0.693**	0.639**	– 0.232**	0.553**	
10	Outgroup stereotypes	4.04	1.13	– 0.729**	– 0.746**	– 0.822**	– 0.704**	0.750**	– 0.717**	0.331**	– 0.552**	– 0.763**

**p < .0.01, *p < .0.05.

### Manipulation check

An ANOVA on the felt understanding manipulation (felt understanding: understood vs. misunderstood) check confirmed a felt understanding main effect (*F* (1,473) = 58.57, *p* < 0.01, η_p_^2^ = 0.110). The manipulation of felt understanding yielded the intended effect, with felt understanding being higher in the understood condition (*M* = 4.30, *SD* = 1.18) than in the misunderstood condition (*M* = 3.51, *SD* = 1.05).

### MANOVA of intergroup orientations

Table 2 shows the descriptive statistics for each scale based on the felt understanding condition, as well as the inferential statistics and effect size estimates from the MANOVA. The analysis of intergroup orientations revealed a multivariate main effect of felt understanding (λ = 0.93, *F* (3, 467) = 11.42, *p* ≤ 0.01, η_p_^2^ = 0.068). The univariate main effect of felt understanding was significant for the three outcome variables (*F*s(1, 469) ≥ 18.92, *p*s ≤ 0.01, η_p_^2^ ≥ 0.039). Perceptions of the intergroup relationship (*M* = 3.97, *SD* = 1.22 vs. *M* = 3.34, *SD* = 1.16), outgroup trust (*M* = 3.97, *SD* = 0.97 vs. *M* = 3.54, *SD* = 0.93), and feelings toward the outgroup (*M* = 48.48, *SD* = 23.26 vs. *M* = 39.25, *SD* = 22.77) were all more positive in the understood condition than in the misunderstood condition.

**Table 2:** MANOVA results, and descriptive statistics for each outcome variable.

MANOVA analysis	Main effect of felt understanding	Individual scales	Understood *M*(*SD*)		Misunderstood *M*(*SD*)	
Intergroup orientations	λ = 0.932, *F*(3, 467) = 11.418, *p* < .001, η^2^_p_ = 0.068	Perceptions of intergroup relationship	3.97	(1.22)	3.34	(1.16)
		Outgroup trust	3.97	(0.97)	3.54	(0.93)
		Outgroup feelings	48.48	(23.26)	39.25	(22.77)
Action intentions	λ = 0.949, *F*(3, 472) = 8.454, *p* < .001, η^2^_p_ = 0.051	Avoid	3.51	(1.67)	4.19	(1.67)
		Approach	4.15	(1.35)	3.61	(1.38)
		Confront	2.28	(1.20)	2.21	(1.18)

### MANOVA of action intentions

The analysis of action intentions revealed a multivariate main effect of felt understanding (λ = 0.95, F (3, 472) = 8.45, p < 0.01, η_p_^2^ = 0.051). The univariate main effect of felt understanding was significant for the outcome variables (except for confrontation) (*F*s(1,469) ≥ 18.24, *p*s ≤ 0.01, η_p_^2^ ≥ 0.037). Avoidance (*M* = 3.51, *SD* = 1.67 vs. *M* = 4.19, *SD* = 1.67) and approach (*M* = 4.15, *SD* = 1.35 vs. *M* = 3.61, *SD* = 1.38) were all more positive in the understood condition than in the misunderstood condition.

### Parallel mediational model

**Figure 2:**
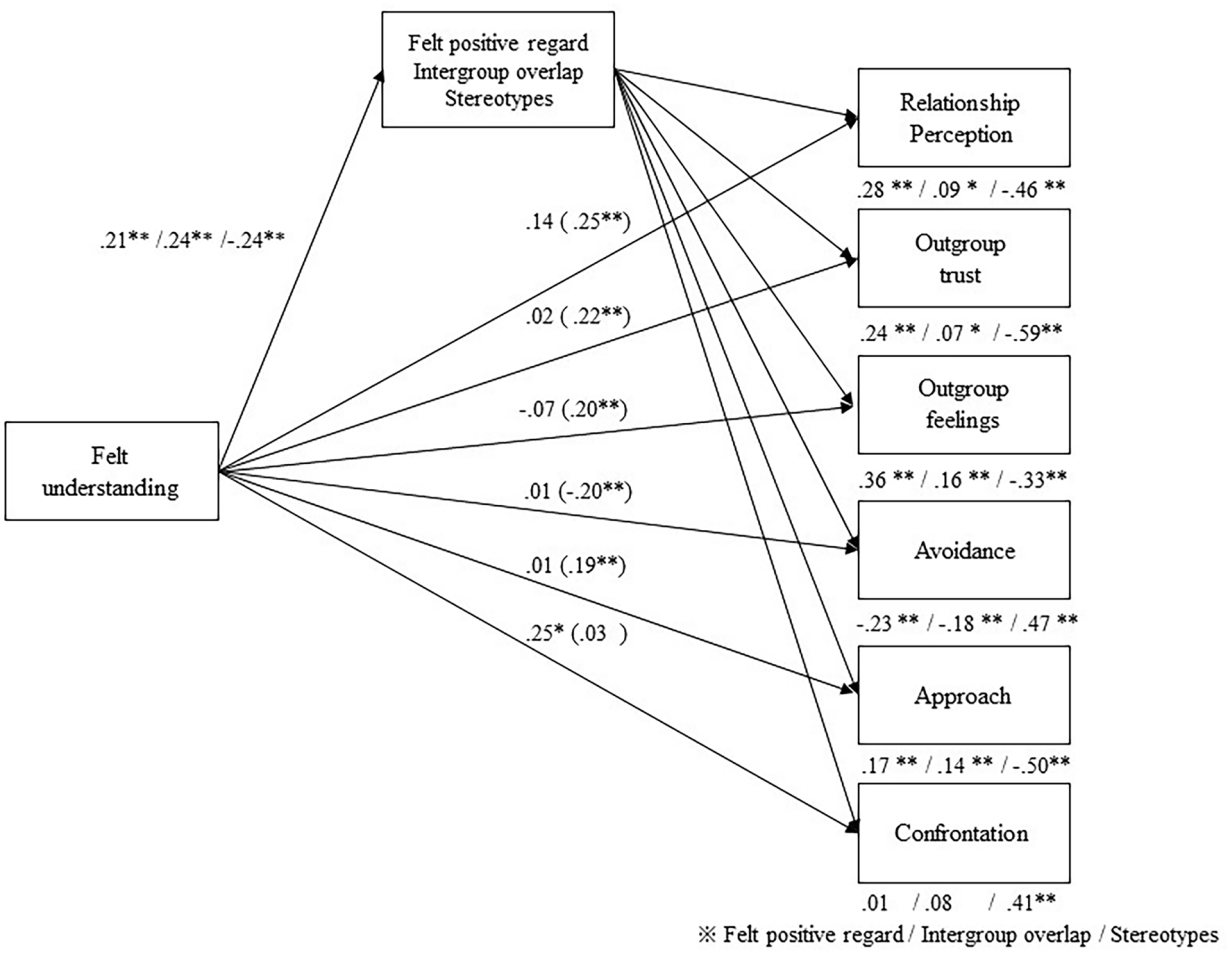
Results of the parallel multiple mediation models.

We hypothesized that three mediators would drive felt understanding processes in intergroup relations: felt positive regard, intergroup overlap, and outgroup stereotypes. Table 3 and Fig. 2 show the results of the parallel multiple mediation models (see online supplementary materials for full estimates).

**Table 3:** Parallel model for indirect effects of felt understanding on intergroup relations through felt positive regard, outgroup stereotype, and intergroup overlap.

Mediator	Bootstrap estimates	*SE*	95% CI lower	95% CI upper
Perception of relationship				
Felt positive regard	**0.059**	0.017	0.031	0.100
Intergroup overlap	**0.022**	0.011	0.004	0.047
Outgroup stereotypes	**0.110**	0.026	0.066	0.166
Total indirect effect	**0.191**	0.033	0.127	0.258
Outgroup trust				
Felt positive regard	**0.051**	0.015	0.025	0.086
Intergroup overlap	**0.017**	0.008	0.003	0.035
Outgroup stereotypes	**0.141**	0.027	0.091	0.199
Total indirect effect	**0.208**	0.037	0.136	0.283
Outgroup feelings				
Felt positive regard	**0.076**	0.020	0.041	0.122
Intergroup overlap	**0.040**	0.013	0.019	0.069
Outgroup stereotypes	**0.079**	0.021	0.043	0.126
Total indirect effect	**0.196**	0.034	0.129	0.261
Avoidance				
Felt positive regard	− **0.048**	0.017	– 0.087	– 0.021
Intergroup overlap	– **0.043**	0.012	– 0.073	– 0.023
Outgroup stereotypes	– **0.112**	0.026	– 0.170	– 0.068
Total indirect effect	– **0.204**	0.035	– 0.271	– 0.137
Approach				
Felt positive regard	**0.036**	0.016	0.010	0.073
Intergroup overlap	**0.036**	0.012	0.017	0.063
Outgroup stereotypes	**0.119**	0.029	0.069	0.185
Total indirect effect	**0.191**	0.034	0.124	0.260
Confrontation				
Felt positive regard	0.003	0.016	– 0.029	0.035
Intergroup overlap	0.021	0.013	– 0.002	0.050
Outgroup stereotypes	– **0.099**	0.024	– 0.151	– 0.059
Total indirect effect	– **0.075**	0.022	– 0.121	– 0.036

Significant values are in bold.

Values are standardized coefficients. *SE* standardized error, *CI* confidence interval.

First, the total indirect effects of felt understanding on intergroup outcomes through felt positive regard, intergroup overlap, and outgroup stereotypes were statistically significant, except for confrontation. Second, the specific indirect effect of felt understanding on intergroup outcomes through felt positive regard was statistically significant across the outcomes, except for confrontation: Felt understanding was positively associated with felt positive regard (β = 0.21, *p* < 0.01), and more felt positive regard was associated with more positive outcomes (β ≥  ± 0.17, *p* < 0.01). Third, the specific indirect effect of felt understanding on intergroup outcomes, except for confrontation through intergroup overlap, was statistically significant: Felt understanding was positively associated with intergroup overlap (β = 0.24, *p* < 0.01), and more intergroup overlap was associated with more positive outcomes (β ≥  ± 0.07, *p*s < 0.05). Fourth, the specific indirect effect of felt understanding on intergroup outcomes through outgroup stereotyping was statistically significant across all outcomes. Felt understanding was negatively associated with outgroup stereotypes (β = − 0.24, *p* < 0.01), and lesser outgroup stereotypes were associated with more positive outcomes (β ≥  ± 0.33, *p*s < 0.05). Thus, the effect of the condition on intergroup outcomes was significantly mediated by felt positive regard, intergroup overlap, and outgroup stereotypes.

### Post hoc test of the sequential mediational model

The parallel mediation model showed that the mediating roles of felt positive regard and intergroup overlap on intergroup outcomes may have been less substantial than those of outgroup stereotypes. In particular, the effects of felt positive regard and intergroup overlap on confrontation were statistically insignificant, and the indirect effects of felt understanding through both were also insignificant. The results showed a pattern consistent with mediation^54^. Although there was no initial hypothesis or preregistration for a direct causal association from felt positive regard and intergroup overlap to outgroup stereotypes, it is reasonable to suggest that enhancing felt positive feelings and intergroup overlap could help diminish outgroup stereotypes. Previous research supports the notion that felt positive regard and intergroup overlap are negatively associated with outgroup stereotyping^19,55–57^, suggesting possible indirect effects of felt positive regard and intergroup overlap on intergroup outcomes. Therefore, to further explore this possibility, we performed a post-hoc test of the sequential model in which we treated the association between felt positive regard or intergroup overlap and intergroup outcomes as causational rather than merely correlational.

Table 4 presents the results of the sequential multiple mediation model. First, all the specific indirect effects of felt understanding on intergroup outcomes through felt positive regard and then outgroup prejudice were statistically significant: Felt understanding was positively associated with felt positive regard (β = 0.21, *p* < 0.01), and more felt positive regard was associated with fewer outgroup stereotypes (β = − 0.65, *p* < 0.01); fewer outgroup stereotypes were associated with more positive intergroup outcomes (β ≥  ± 0.33, *p* < 0.01). Likewise, the specific indirect effects of felt understanding on intergroup outcomes through intergroup overlap and then outgroup stereotypes were statistically significant: Felt understanding was positively associated with intergroup overlap (β = 0.24, *p* < 0.01), and more intergroup overlap was associated with less outgroup stereotypes (β = − 0.18, *p* < 0.01); less outgroup stereotypes were associated with more positive outcomes (β ≥  ± 0.33, *p* < 0.01). Thus, the effect of felt understanding on intergroup outcomes was at least partially sequentially mediated by felt positive regard, intergroup overlap, and outgroup stereotypes.

**Table 4:** Sequential model for indirect effects of felt understanding on intergroup relations through felt positive regard or intergroup overlap, and then outgroup stereotype.

Mediator	Bootstrap estimates	*SE*	95%CI lower	95%CI upper
Perception of relationship				
Via outgroup stereotypes				
Felt positive regard	**0.063**	0.017	0.034	0.099
Intergroup overlap	**0.020**	0.006	0.010	0.036
Outgroup trust				
Via outgroup stereotypes				
Felt positive regard	**0.080**	0.018	0.047	0.117
Intergroup overlap	**0.025**	0.007	0.013	0.043
Outgroup feelings				
Via outgroup stereotypes				
Felt positive regard	**0.045**	0.013	0.023	0.075
Intergroup overlap	**0.014**	0.005	0.007	0.026
Avoidance				
Via outgroup stereotypes				
Felt positive regard	– **0.064**	0.016	– 0.099	– 0.036
Intergroup overlap	– **0.020**	0.006	-0.036	– 0.010
Approach				
Via outgroup stereotypes				
Felt positive regard	**0.067**	0.018	0.037	0.106
Intergroup overlap	**0.021**	0.018	0.011	0.038
Confrontation				
Via outgroup stereotypes				
Felt positive regard	– **0.056**	0.016	– 0.093	– 0.030
Intergroup overlap	– **0.017**	0.006	– 0.033	– 0.009

Significant values are in bold.

Values are standardized coefficients. *SE* standardized error, *CI* confidence interval.

## Discussion

This study investigated whether and how felt understanding can shape intergroup relationships. Although there is emerging evidence of the benefits of felt understanding in intergroup relations^1,9–11^, little is known about the processes through which felt understanding has favorable effects. We tested the main hypothesis that felt understanding positively affects intergroup outcomes such as intergroup trust by increasing felt positive regard (Hypothesis 1), intergroup overlap (Hypothesis 2), and outgroup stereotypes (Hypothesis 3). The results of this study support these hypotheses in the context of Japanese and Chinese relations. The results of parallel mediation analyses^22^ provided evidence that felt positive regard, intergroup overlap, and outgroup stereotypes mediated the associations between felt understanding and intergroup outcomes, such as perceptions of intergroup relationships, outgroup feelings, outgroup trust, avoidance, and approach. Further, the results of post hoc sequential mediation analyses provided tentative evidence that the associations between felt understanding and intergroup outcomes are mediated by felt positive regard and intergroup overlap, and then by outgroup stereotypes.

Consistent with Livingstone et al.^11^, the findings demonstrated the effect of felt understanding on intergroup outcomes in Japanese and Chinese relations. The manipulation of felt understanding had the expected effect: feeling understood rather than misunderstood led to more positive intergroup orientations and action intentions. However, felt understanding did not affect one of the action intentions, confrontation, which is inconsistent with the findings of Livingstone et al.^11^. This could be due to naïve dialecticism or causal attributions to external situations. East Asians have a flexible self-concept and are open to contradictions^40,41^, and are less likely to respond negatively to others when they do not feel understood^9^. Indeed, in a survey of a Japanese sample, Ioku and Watamura^9^ found that Japanese respondents did not want to confront the Chinese in Japan when they thought that they did not understand them. Despite these differences, the results of the present study are generally consistent with those reported by Livingstone et al.^11^. Our findings from East Asia provide additional experimental evidence for the notion that felt understanding includes the second-order theory of mind^7,9,11^, which is known to be comparatively universal^58,59^.

The findings regarding the indirect effect of felt understanding through felt positive regard in intergroup contexts are consistent with the perspective of positive regard for interpersonal relations^13–15^. This perspective suggests that feeling understood by others satisfies the need for positive regard, thereby increasing positive orientations and action intentions toward an outgroup. When the Japanese respondents felt that the Chinese understood their beliefs, they displayed positive attitudes and action intentions by increasing their perceived positive regard. This is noteworthy because many studies have shown that East Asians may have a lower need for positive regard than Westerners^35–37^.

We also obtained evidence of the indirect effect of felt understanding through intergroup overlap in intergroup contexts, consistent with perspective-taking research and theory^20–22^. Specifically, Goldstein et al.^22^ proposed that being on the receiving end and taking another’s perspective are similar in that the two brains merge. Our data from East Asia support this proposition, but the indirect effect via intergroup overlap is weaker than that of the other mediating variables. There are two possible explanations for these results. First, as described in the Introduction, this may be due to the cultural characteristics of East Asians: tolerance of contradictions^9,40,41^. That is, when the Japanese thought that the Chinese understood them, they might have thought that, “They are us, but differ somehow,” because East Asians tend to tolerate contradictions. This subtle sense may obscure the process of intergroup overlap from the benefits of felt understanding of intergroup relations. Second, there may be a sequential rather than parallel association between intergroup overlap (and felt positive regard) and outgroup stereotypes. When the Japanese thought that the Chinese understood them, they might have thought that the Japanese and Chinese were similar and might have reduced their stereotypes. The results of the sequential mediation analysis support this interpretation, which is consistent with previous studies^56,57^. However, this is a tentative interpretation and requires further research.

Furthermore, evidence of the indirect effect of felt understanding through outgroup stereotypes in intergroup contexts is consistent with the wealth of research on the norm of reciprocity^26–29^. According to the norm of reciprocity, humans tend to repay pleasant treatment while retaliating against unpleasant treatment^26,27^. In line with this, when Japanese respondents perceived that Chinese people understood their perspectives, they reduced their negative stereotypes, showing positive orientations and action intentions toward the Chinese. The findings extend the research on the norm of reciprocity to reveal that outgroup stereotypes play a consistent mediating role when felt understanding affects intergroup relations.

Although this study contributes to our understanding of why felt understanding benefits intergroup relationships, it has several limitations. First, the indirect effect of felt understanding on intergroup outcomes through intergroup overlap seemed weaker than that of the others across the six outcomes. We attribute this result to cultural factors, the tolerance of contradictions in East Asia, or a sequential association of intergroup overlap with outgroup stereotypes. If the former is true, then intergroup overlap may not mediate the positive effect of felt understanding of intergroup relations with another sample from East Asia, such as Korea, or to a lesser extent. If the latter is true, the positive effect of felt understanding on intergroup relations may be mediated by intergroup overlap and then outgroup stereotypes in samples from East Asia, the US, and the UK. Testing these possibilities reveals the role of intergroup overlap in the mechanisms of felt understanding.

Second, there is an alternative explanation for Hypothesis 2: In our study, participants comprised the majority group (Japanese vs. Chinese in Japan). A higher status in intergroup relations may have affected the process of feeling positive regard. More specifically, because people from the majority tend to pay less attention to others than their counterparts from the minority^60–62^, less attention to the Chinese may have made the mediating effect of intergroup overlap unclear. This alternative possibility can be investigated, for example, using a sample of heterogeneous people in relation to transgender and gender diversity.

Third, readers should be cautious about inferring mediation, especially causal mediation, from the mediator to the outcome paths. This study manipulated a single independent variable, measured the mediators and outcomes, and tested the indirect effects of the independent variable using mediators. This type of design is called a measurement-of-mediation design^63^. Several scholars have criticized such approaches and proposed causal chain designs in which both the manipulation and measurement of the mediators of interest are feasible^63–65^. This approach allows researchers to draw stronger inferences regarding the causal relationships between variables. For example, a recent study on felt understanding investigated the effects of felt positive regard on intergroup outcomes with a causal chain design after confirming the mediating effect of felt positive regard between felt understanding and intergroup outcomes using a measurement-of-mediation design^12^. Thus, future research adopting causal chain designs that manipulate and measure mediators will strengthen the causal inferences of our findings.

## Conclusion

The present findings support the mediating roles of felt positive regard, intergroup overlap, and outgroup stereotypes in the benefits of felt understanding in intergroup relations with respect to Japanese and Chinese relations in Japan. The results of this study suggest that felt understanding affects more positive intergroup outcomes (intergroup relationship perceptions, outgroup feelings, outgroup trust, avoidance, and approach) because it increases felt positive regard and intergroup overlap and decreases outgroup stereotypes. Thus, the findings extend research on felt understanding in intergroup relations by indicating that the benefits of felt understanding in intergroup relations from East Asia may well be attributed in part to its effect on the positive evaluation of “they liked us,” the shared identity of “they are us,” and the norm of “returning favor.”

### Supplementary Information

Supplementary Information.

## Data Availability

Data and materials for this study are available on the OSF website: https://osf.io/846h9/?view_only=68ff62c5477144deb2afb5154375659f.
