# Supplementary material for: An experimental study of the process of felt understanding in intergroup relations: Japanese and Chinese relations in Japan
Source: Sci Rep. 2024 Jun 7;14:13096. doi: 10.1038/s41598-024-63227-0 (PMC11161610; doi:10.1038/s41598-024-63227-0)
Supplement: Supplementary file 1 — Supplementary Information. [file 41598_2024_63227_MOESM1_ESM.docx]

SUPPLEMENTARY MATERIAL

Contents

[Appendix 1. Full Estimates of Parallel Model for the Effects of Felt Understanding on Intergroup Relations Through Felt Positive Regard, Outgroup Stereotype, and Intergroup Overlap. 2](#_Toc163565360)

# Appendix 1. Full Estimates of Parallel Model for the Effects of Felt Understanding on Intergroup Relations Through Felt Positive Regard, Outgroup Stereotype, and Intergroup Overlap.
